# Pericardial effusion in children at tertiary national referral hospital, Addis Ababa, Ethiopia: a 7-year institution based review

**DOI:** 10.1186/s12873-023-00922-7

**Published:** 2024-01-07

**Authors:** Selamawit Amare, Henok Tadele

**Affiliations:** 1 Department of Pediatrics and Child Health, Yekatit 12 Medical College, Addis Ababa, Ethiopia; 2 Department of Pediatrics and Child Health, School of Medicine, College of Health Sciences, Addis Ababa University, Addis Ababa, Ethiopia

**Keywords:** Pericardial effusion, Purulent effusion, Tuberculous effusion, Pericardial tamponade, Children, Ethiopia, Africa

## Abstract

**Background:**

Pericardial effusion (PE) is a rare yet an important cause of child mortality due to collection of excess fluid in pericardial space. The study aimed to describe the PE profile in the national cardiac referral hospital, Addis Ababa, Ethiopia.

**Methods:**

The study employed cross-sectional study design for a 7-year review of childhood PE in Tikur Anbessa Specialized Hospital. Descriptive and analytic statistics were applied.

**Results:**

There were 17,386 pediatric emergency/ER admissions during the study period, and PE contributed to 0.47% of ER admissions. From 71 included subjects, 59% (42) were males with mean age of 7.8 ± 3.3 years. Cough or shortness of breath,73.2% (52) and fever or fast breathing, 26.7% (19), were the common presenting symptoms. The median duration of an illness before presentation was 14days (IQR: 8–20). The etiologies for pericardial effusion were infective (culture positive–23.9%, culture negative–43.6%, tuberculous-4.2%), hypothyroidism (4.2%), inflammatory (12.7%), malignancy (7%) or secondary to chronic kidney disease (1. 4%). Staphylococcus aureus was the most common isolated bacteria on blood culture, 12.7% (9) while the rest were pseudomonas, 7% (5) and klebsiella, 4.2% (3). Mild, moderate and severe pericardial effusion was documented in 22.5% (16), 46.5% (33), and 31% (22) of study subjects, respectively. Pericardial tamponade was reported in 50.7% (36) of subjects. Pericardial drainage procedure (pericardiocentesis, window or pericardiotomy) was performed for 52.1% (37) PE cases. The case fatality of PE was 12.7% (9). Pericardial drainage procedure was inversely related to mortality, adjusted odds ratio 0.11(0.01–0.99), *p* 0.049).

**Conclusion:**

PE contributed to 0.47% of ER admissions. The commonest PE presentation was respiratory symptoms of around two weeks duration. Purulent pericarditis of staphylococcal etiology was the commonest cause of PE and the case fatality rate was 12.7%. Pericardial drainage procedures contributed to reduction in mortality. All PE cases should be assessed for pericardial drainage procedure to avoid mortality.

## Background

Pericardial effusion (PE) is a serious condition where fluid accumulates in the pericardial sac. Unless diagnosed early and treated it could result in severe cardiovascular compromise leading to death through a phenomenon called cardiac tamponade [1, 2]. Half of PE cases are idiopathic in developed settings while the remaining are caused by infectious, cancer, iatrogenic and inflammatory etiologies [2–6]. PE is largely of infectious origin in developing countries, mostly tuberculosis (TB) and bacterial etiologies like staphylococcus [7–11].

Chest pain, dyspnea on exertion, orthopnea and fever are common PE symptoms. Physical examination could be normal in a patient without any cardiovascular derangement. Tachycardia, raised jugular venous pressure, muffled and distant heart sounds, hypotension and pulsus paradoxus are eminent signs of cardiac tamponade [1, 2]. Diagnosis of PE is easily made by echocardiography. Echocardiographically, PE is graded into mild (< 10 mm), moderate (10-20 mm) and severe (> 20 mm) based on size [2]. Echocardiography also helps to assess presence or absence of cardiac tamponade. Right atrial free wall collapse during systole, right ventricular free wall collapse during diastole, inferior vena cava plethora, and exaggerated inflow respiratory cycle changes in atrioventricular valves are some of the echocardiographic features of cardiac tamponade [1, 12, 13]. Pericardial fluid can be subjected for chemistry, cytology, microbiologic assays (acid fast bacilli, gram stain, culture) [13].

Any patient with cardiac tamponade, suspicion of bacterial or neoplastic causes should undergo pericardiocentesis. After stabilization of the patient further investigation and treatment are decided based on an underlying cause for PE [1, 2, 13]. When traumatic PE is suspected either sternotomy or pericardial window is indicated while pericardiocentesis or indwelling catheter is used for nontraumatic PE [13].

Timely pericardiocentesis or pericardial drainage procedure was reported to be safe and ascribed to decrease mortality among subjects with PE with hemodynamic compromise [9, 14, 15]. Diagnosis of tuberculosis PE [16], staphylococcal PE [4], younger age with solid tumor diagnosis and need for pericardial fluid drainage [5] were associated with high mortality. PE among children is an under studied subject with very limited reports from Africa and none from Ethiopia. Hence, the study aimed to assess the clinico-etiologic profile of PE and determinants of its treatment outcome among PE subjects admitted to the national cardiac referral center in Ethiopia.

## Methods

### Study area

This study was conducted in Addis Ababa University, College of Health Sciences, Tikur Anbessa Specialized Teaching Hospital (TASH), Department of Pediatrics and Child Health, Addis Ababa, Ethiopia. TASH is the largest and national cardiac referral center for Ethiopia. Pediatric cardiac surgical interventions including pericardial drainage procedures are provided by cardiothoracic surgeons and pediatric cardiologists. The hospital has dedicated well-equipped cardiac intensive care unit with various monitoring facilities, echocardiography, cardiac catheterization and dedicated cardiothoracic operation theatre rooms. Pericardial window and pericardiotomy are done by cardiothoracic surgeons while pericardiocentesis are performed by pediatric cardiologists.

### Study design

The study employed cross-sectional study design for the review of medical records of 71 PE subjects who were admitted to TASH between April 2015 and May 2022. Demographic, clinical and echocardiographic data were collected from the medical records.

### Source population

All children, aged 1-month to 15-year, with the diagnosis of pericardial effusion and admitted to TASH.

### Inclusion and exclusion criteria

All children, aged 1-month to 15-year, with the diagnosis of pericardial effusion and admitted to TASH were included in the study. PE cases with incomplete medical records were excluded from the study.

### Study variables

The dependent variable was hospital discharge outcome of PE subjects: alive or dead. Age, sex, residence, chief compliant, duration of illness, admission pulse rate, admission respiratory rate, admission temperature, admission oxygen saturation, severe acute malnutrition, any comorbidities, tamponade clinical signs, associated foci of infection, severity of PE on echocardiography, presence of cardiac tamponade on echocardiography, and any pericardial drainage procedure were independent variables.

### Operational definitions

PE was graded into mild, moderate and severe when PE measured < 10 mm, 10-20 mm and > 20 mm on echocardiography, respectively [2]. Cardiac tamponade was considered when right atrial free wall collapse during systole, right ventricular free wall collapse during diastole, inferior vena cava plethora, and exaggerated inflow respiratory cycle changes in atrioventricular valves were evident on echocardiography [1]. Admission vital signs were assessed as normal or abnormal based on pediatric advanced life support guideline [17]. Severe acute malnutrition/SAM was considered when weight for age was <-3 Z score on World Health Organization (WHO) growth curves, bilateral pitting edema or low mid upper arm circumference (MUAC) was present in an under five children [18]. In children above 5-year of age, severe thinness or SAM was considered when Body Mass Index/BMI for age was below − 3 standard deviations on WHO growth curves [19]. Clinical purulent PE was considered when a pus was drained from pericardial space. Treatment outcome was a hospital discharge outcome of a PE subject: death or survival.

### Data analysis

Data analysis was done using statistical software for social sciences version 25. Univariate association with the dependent variable was assessed using chi-square test. A variable with *p value* less than 0.05 was selected for final binary multivariable logistic regression model. Previously studied variables (age and comorbid conditions) were included in the final model [5]. *P* value less than *0.05* was set as significant and association was reported using odds ratio with its 95% confidence interval.

## Results

### Sociodemographic profile

Medical records of 81 PE subjects were retrieved and 10 were excluded from the study due to incomplete data. There were 17,386 pediatric emergency admissions during the study period, and PE contributed to 0.47% of ER admissions. Out of the 71 subjects included in the study majority were males,59% (42). The mean and median age of study subjects were 7.8 ± 3.3 years, and 8 years (Interquartile range/IQR: 6–11), respectively. The majority, 59% (42), came from the regional states of Ethiopia, out of Addis Ababa. (See Table 1)

**Table 1:** Sociodemographic and clinical variables association with treatment outcome of pericardial effusion

Variables	Category	alive	Died	COR (95%CI)	*P* value
Age(years)	≤5	15	4	2.51(0.59-10.55)	0.21
	>5	47	5	1	
Sex	Male	38	4	0.51(0.12-2.07)	0.34
	Female	24	5	1	
Residence	Addis Ababa	25	4	1.18(0.29-4.85)	0.81
	Other regions	37	5	1	
Clinical presentation	Fever/fast breathing	17	2	0.76(0.14-4.01)	0.74
	Cough/Shortness of breath	45	7	1	
Duration of an illness in days	<10	18	2	0.69(0.13-3.69)	0.67
	≥10	44	7		
Admission pulse rate	Normal	10	2	1.49(0.27-8.22)	0.65
	Elevated	52	7	1	
Admission respiratory rate	Normal	5	2	3.26(0.53-20.06)	0.20
	Elevated	57	7	1	
Admission Temperature	Normal	5	2	3.26(0.53-20.06)	0.20
	Hypothermia / Febrile	57	7	1	
Admission oxygen saturation	Hypoxia	37	6	1.35(0.31-5.9)	0.69
	Normal	25	3	1	
Severe acute malnutrition	Yes	11	2	1.32(0.24-7.26)	0.75
	No	51	7	1	
Any comordities	No	48	5	0.36(0.09-1.54)	0.17
	Yes	14	4	1	
Primary focus of infection	Pneumonia	40	5	0.69(0.17-2.83)	0.60
	Pyomyositis/osteomyelitis/others	22	4	1	
Pericardial effusion size on echocardiography	Mild	14	2	3(0.25-35.32)	0.39
	moderate	27	6	4.67(0.52-41.8)	0.17
	Severe	21	1	1	
Echocardiographic report of cardiac tamponade	Yes	30	6	2.13(0.49-9.3)	0.31
	No	32	3	1	
Pericardial drainage procedure	Yes	36	1	0.09(0.01-0.77)	0.02*
	No	26	8	1	

### Clinical profile

Fever or fast breathing was the presenting symptom in 26.7% (19) of study subjects while cough or shortness of breath was the most common chief complaint, 73.2% (52). The median duration of an illness before presentation to a hospital was 14days (IQR: 8–20). Deranged vital sign evidenced by tachycardia, tachypnea, fever, and hypoxia was present in 86% (61), 90% (64), 90% (64) and 59% (42) of study subjects, respectively. Only 18.3% (13) had severe acute malnutrition. Majority didn’t have any associated comorbidities, 73.2% (52). Associated co-morbidities included auto-immune disease (9.9%), hypothyroidism (4.2%), malignancy (7% - two cases of leukemia and three cases of lymphoma) and chronic renal failure (1.4%). Majority of subjects with comorbid conditions had a duration of illness that lasted for over 10days before presentation, 20% (14). Pneumonia, 63.4% (45), was the most common primary site of infection while pyomyositis and osteomyelitis, 36.7% (26), were the other foci of infection. Clinical evidence of pericardial tamponade was documented in 53.5% (38) study subjects: distended neck veins 12.7% (9), distant heart sounds 9.9% (7), and combination of distant heart sounds and distended neck veins were present in 31% (22) of the study subjects. Leukocytosis was found in 64.8% (46) of study subjects. Elevated Erythrocyte Sedimentation Rate (ESR) was documented in 97.2% (69) while a raised C reactive protein (CRP) was evident in 83.1% (59) of study subjects. (See Table 1)

From the etiologic agents for PE: 23.9% (17) purulent, 12.7% (9) inflammatory, 7% (5) malignancy, tuberculosis 4.2% (3), hypothyroidism 4.2% (3), 1.4% (1) renal/chronic kidney disease, and 43.6% (31) clinical purulent PE with no identifiable microorganism were documented. Apart from infectious causes, only three subjects with lymphoma had severe PE while all others had mild or moderate PE. Out of 48 documented blood culture results, staphylococcus aureus was the most common isolated bacteria, 12.7% (9). Pseudomonas, 7% (5) and klebsiella, 4.2% (3) were the other isolated bacteriological agents while the remaining didn’t show any growth. From pericardial fluid, gram stain positive bacteria were detected in 16.9% (12) while staphylococcus aureus was detected in pericardial fluid culture in 4.2% (3) of study subjects. From pericardial fluid analysis or pericardial biopsy report, acute inflammation, 26.8% (19); chronic inflammation, 12.7% (9); and tuberculosis, 4.2% (3) were detected. One case of tuberculosis also had positive GeneXpert from pericardial fluid. There was only one report of HIV infection among PE subjects.

Mild, moderate and severe pericardial effusion was documented in 22.5% (16), 46.5% (33), and 31% (22) of the study subjects, respectively. Pericardial tamponade was evident in 50.7% (36) of PE subjects. Pericardial drainage procedure (pericardiocentesis, window or pericardiotomy) was done in 52.1% (37) and majority had severe or moderate PE category, 49.2% (35). Only one subject had pericardiocentesis /ultrasound guided pericardial drainage. Mild 2.8% (2), moderate 19.7% (14) and severe 29.6% (21) PE were documented among those who had the pericardial drainage procedure. None of the study subjects who had undergone pericardial drainage procedure developed procedure related complication.

There was no report of structural heart disease in any of PE subjects on echocardiography. Different combination of antimicrobials, anti-tuberculous therapy, steroids, chemotherapy and thyroxine were used in addition to the surgical management in the treatment course of PE subjects. The median duration of hospitalization was 20 days (IQR: 14–28). Majority were discharged alive, 87.3% (62). The case fatality of PE was 12.7% (9). Only pericardial drainage procedure showed association with PE treatment in univariate analysis (See Table 1).

Pericardial drainage procedure was inversely related with mortality among PE subjects, adjusted odds ratio 0.11(0.01–0.99) *p* = 0.049). (See Table 2)

**Table 2:** Factors associated with pericardial effusion treatment outcome

Variables	Category	Outcome		COR (95%CI)	Adjusted OR (95% CI)	*P* value
		Survived	Died			
Age (years)						
	< or equal to 5	15	4	2.51(0.59-10.55)	2.21(0.47-10.27)	0.313
	>5	47	5	1	1	
Comorbid conditions						
	No	48	5	0.37(0.09-1.54)	1.54(0.32-7.36)	0.588
	Yes	14	4	1	1	
Pericardial drainage procedure						
	Yes	36	1	0.09(0.01-0.77)	0.11(0.01-0.99)	0.049*
	No	26	8	1	1	

## Discussion

We report pericardial effusion among children from the national cardiac referral center in Ethiopia. We analyzed the clinical presentation, treatment outcome and its determinants using a 7-year review. PE contributed to 0.47% of pediatric emergency admissions. Pyogenic PE was the dominant type while tuberculosis PE was documented in only three subjects. Pericardial drainage procedure was inversely related to mortality among PE subjects.

PE or pericardial tamponade contributed to less than 1% of pediatric emergency admissions in our study. This is in agreement with reports from other countries [10, 20]. This underscores the rarity of PE or pericardial tamponade in children. The mean age at PE presentation was 7.8 ± 3.3 years in our study. This is in agreement with reports from Nigeria, Iran, Turkey and India [9, 10, 15, 21, 22]. This is as opposed to a younger age at presentation in another Turkish study [23]. A smaller number of study subjects and focus on purulent PE in the later study could be ascribed for the noted difference. Half of PE subjects in our study had pericardial tamponade. This could be due to the referral pattern where subjects with severe PE or suspiciously severe PE requiring further assessment and possible pericardial drainage were referred to our national cardiac center.

In this study, cough or shortness of breath was the most common PE presentation followed by fever or fast breathing. This is in line with other studies [4, 8, 15, 20–22]. The median duration of illness of PE subjects was about two weeks in this study. This is comparable to reports from other studies [15, 20]. This enlightens the acute PE presentation as a case necessitating a hospital admission as a primary disease or as an associated illness. Distended neck veins and distant heart sounds were evident in subjects with tamponade effect in this study. This is consistent with other reports [4, 9, 22]. There is a need for meticulous assessment to detect tamponade among subjects with PE.

Comorbid conditions were documented in very few study subjects in the current study. The few documented comorbid conditions of autoimmune, malignancy, hypothyroidism and chronic kidney disease could be explained by the high chances detecting PE cases in their respective chronic follow up clinic of their primary diseases in our set up as the study setting hospital is the national referral hospital where multiple specialties and sub-specialties care are provided. Lung, joint and muscle infections were concomitantly documented among our study subjects with PE. These are in agreement with previous reports [15, 21–23]. The findings underline a need for comprehensive assessment for PE among children with these sites of primary infection even in the absence of a comorbid conditions.

Purulent PE, clinical or laboratory confirmed, was the most common form in our study. And, staphylococcus was the most isolated bacteriologic agent. This is in harmony with previous reports from developing countries [8, 15, 21]. This contrasts with studies from other developing countries where mycobacterium tuberculosis was the common etiologic agent [9–11, 22, 23]. Whereas Ethiopia is a TB endemic country our findings should be interpreted in light of the short duration of an illness among our study subjects with a median duration of illness of about two weeks. It is expected that subjects with tuberculosis have protracted symptoms. Clinical purulent PE with no identifiable etiologic agent could be due to antibiotic exposure before arrival to our referral set up limiting the yield of bacteriologic test.

A little over one tenth of PE subjects had died in this study. This is in agreement with other studies [10, 15, 21, 23]. Pericardial drainage procedure reduced mortality among PE subjects in our study. This is similar to other studies [10, 21]. However, a reverse was documented in another study [11]. In this study, significant number of deaths happened at home after hospital discharge and majority had pericardiocentesis done earlier, it could be due to recollection and a missed opportunity for life saving pericardiocentesis. This was also stressed by the authors. Our finding cements the established evidence of the lifesaving effect of a pericardial drainage procedure among PE subjects. Hence, timely clinical and echocardiographic assessment is needed to guide the need for this treatment.

The strength of our study is a review of a good number of years of data on the rare disease. Our limitations include failure to uncover more data for detailed analysis due to the retrospective nature of the study and a single center study. Moreover, a single patient could have a single or combination of indications for pericardial drainage procedure and given the retrospective nature of the study, we couldn’t pinpoint a single indication. Additionally, we couldn’t ascertain whether the mild and moderate cases who had undergone pericardial drainage procedures also had pericardiocentesis done at a referring health facility. However, this study gives the clinic-epidemiologic profile of PE in a Sub-Saharan setting and helps to guide the emergent cardiac care among PE subjects. It also calls for the institution of comprehensive and timely assessment for PE using ultrasound or echocardiography machine in a related setting. It will also serve as a baseline resource for further studies.

## Conclusion

PE contributed to 0.47% of pediatric emergency admissions. Respiratory symptoms with a median duration of illness of two weeks were the common presenting features of PE. Purulent PE, primarily staphylococcal, was the most common form. A little over one-tenth of PE subjects died and mortality was inversely related to the pericardial drainage procedure. All PE cases should be assessed for pericardial drainage procedures to avoid mortality.

## Data Availability

The datasets analyzed during this study are available from the corresponding author on reasonable request.

## Abbreviations

aOR: Adjusted odds ratio
BMI: Body Mass index
COR: Crude odds ratio
CRP: C reactive protein
ER: Emergency room
ESR: Erythrocyte sedimentation rate
HIV: Human Immunodeficiency Virus
IQR: Interquartile range
PE: Pericardial Effusion
SAM: Severe Acute Malnutrition
TASH: Tikur Anbessa Specialized Hospital
TB: Tuberculosis
WHO: World Health Organization
